# Update on the Crosstalk Between Adipose Tissue and Mineral Balance in General Population and Chronic Kidney Disease

**DOI:** 10.3389/fped.2021.696942

**Published:** 2021-08-06

**Authors:** Vasiliki Karava, Athanasios Christoforidis, Antonia Kondou, John Dotis, Nikoleta Printza

**Affiliations:** ^1^Pediatric Nephrology Unit, 1st Department of Pediatrics, Hippokratio General Hospital, Aristotle University of Thessaloniki, Thessaloniki, Greece; ^2^Pediatric Endocrinology Unit, 1st Department of Pediatrics, Hippokratio General Hospital, Aristotle University of Thessaloniki, Thessaloniki, Greece

**Keywords:** calcitriol, parathormone, FGF23, adipose tissue, adipokine, adiponectin, leptin, chronic kidney disease-mineral and bone disease

## Abstract

Adipose tissue is nowadays considered as a major endocrine organ, which apart from controlling lipid metabolism, displays a significant role in energy expenditure, food intake and in the regulation of various systemic physiological processes. Adipose derived pro-inflammatory cytokines and adipokines, particularly leptin and adiponectin, provide inter-communication of adipose tissue with various metabolic pathways, ultimately resulting in a complex network of interconnected organ systems. Recent clinical and experimental research has been focused on exploring the direct interaction between adipokine profile and elements of mineral metabolism, including parathormone (PTH), fibroblast growth factor-23 (FGF23) and calcitriol. The emerging crosstalk between adipose tissue and calcium and phosphorus homeostasis suggests that metabolic disorders from one system may directly affect the other and *vice versa*. It is current knowledge that fat metabolism disturbance, commonly encountered in obese individuals, influences the expression of calciotriopic hormones in general population, while various clinical trials attempting to successfully achieve body fat loss by modulating mineral profile have been published. In chronic kidney disease (CKD) state, there is an increasing evidence suggesting that mineral disorders, influence adipose tissue and linked endocrine function. On the contrary, the impact of disturbed fat metabolism on CKD related mineral disorders has been also evocated in clinical studies. Recognizing the pathogenetic mechanisms of communication between adipose tissue and mineral balance is critical for understanding the effects of metabolic perturbations from the one system to the other and for identifying possible therapeutic targets in case of disrupted homeostasis in one of the two connected systems. To that end, this review aims to enlighten the recent advances regarding the interplay between mineral metabolism, fat mass and adipokine profile, based on *in vitro, in vivo* and clinical studies, in general population and in the course of CKD.

## Introduction

It is current knowledge that white adipose tissue serves as a principal human energy repository in the form of triglycerides, and coordinates lipid metabolism in order to maintain whole body free fatty acid balance. Among the adipocyte-derived secreted proteins, also called adipokines, adiponectin boosts lipid storage and adipogenesis, while leptin blocks adipogenesis and enhances triglyceride hydrolysis (1). Both hormones stimulate fatty acid beta oxidation, inhibiting ectopic fat deposition (1). Emerging evidence indicates that adipokines exhibit not only autocrine and paracrine but also pleiotropic endocrine activity. Leptin and adiponectin intercommunicate with central nervous system, though reciprocal hypothalamic effects, providing homeostasis of energy expenditure, appetite, and subsequently body weight (2). Moreover, adipose tissue derived pro-inflammatory cytokines and adipokines are involved in the regulation of various physiological processes, such as local and systemic inflammation (3), cardiovascular function (4), glucose homeostasis (5), bone hematopoiesis (6), renin-angiotensin system and sodium balance (7). Components of these systems are, in turn, interconnected with elements of mineral homeostasis (8–10). Nevertheless, emerging evidence indicate, apart from indirect, a reciprocal direct communication between mineral metabolism, adiposity and related endocrine function (11). Chronic kidney disease (CKD) is a condition characterized by mineral disorders, which potentially modulate fat metabolism. On the opposite, body fat mass and adipokine profile affects mineral metabolism in general population and may additionally affect CKD-related mineral disorders. The purpose of this review paper is to enlighten the recent advances regarding the direct crosstalk between mineral metabolism, fat mass and adipokine profile, in general population and in CKD, based on *in vitro, in vivo* and clinical studies.

## Adipokines Balance and Mineral Homeostasis in General Population and CKD

### Adipokines Balance in General Population and CKD

In general population, serum adipokines levels are principally influenced by adipose tissue mass and individual energy demands. In specific, adiponectin expression is enhanced in lean individuals, while leptin expression increases with obesity (12, 13). Low energy expenditure state, including fasting and caloric deprivation diets, is significantly linked to decreased serum leptin and possibly to increased serum adiponectin (14).

CKD is associated with perturbated adipokine profile. Reduced renal clearance of leptin and adiponectin, although the latter is primarily excreted by the liver, may in part explicate the higher serum leptin and adiponectin levels commonly observed in both adult and pediatric patients, compared to healthy controls (15–23). Moreover, body fat mass plays, as expected, a major role on adipokines expression among CKD patients (24, 25). In detail, relatively higher serum leptin (15, 24, 25) and relatively lower serum adiponectin levels (26) are often encountered in overweight adult and pediatric patients, while poor nutritional status is often related to lower leptin and higher adiponectin serum profile (27, 28). Furthermore, uremic condition may affect adipokines production (20, 29). Experimental studies indicated that uremic milieu increased adipocyte production of adiponectin and leptin (30–32). In clinical studies, serum leptin levels were positively correlated to various serum inflammatory cytokines levels in CKD 5 patients, suggesting a possible contributive role of systemic inflammation on leptin overproduction in CKD (33, 34). However, other researchers report that adipose tissue leptin and adiponectin expression are downregulated in CKD, as a result of a negative feedback regulation from reduced renal clearance (19, 35, 36).

### Mineral Homeostasis in General Population and CKD

Mineral metabolism is based on a complex network of interconnected organs including intestine, kidney and parathyroid gland, ensuring homeostasis of calcium, phosphate, vitamin D, parathormone (PTH), and fibroblast growth factor-23 (FGF23). Each regulator of mineral metabolism possesses multiple roles. In brief, PTH secretion inhibits phosphate reabsorption in renal proximal tubule, promotes calcium reabsorption in the distal tubules, stimulates renal calcitriol production and increases calcium and phosphate resorption from bone (37). Osteocyte and osteoblast released FGF23 downregulates phosphate reabsorption in renal proximal tubule, activates calcium reabsorption in distal tubules, decreases serum calcitriol, by both suppression of renal 1a-hydroxylase synthesis and stimulation of 24-hydroxylase, and weakly inhibits PTH excretion (38). Both actions on kidney and parathyroid gland are mediated *via* requisite linkage of FGF23 with its co-receptor and co-factor transmembrane protein called Klotho (39). Finally, calcitriol stimulates intestinal calcium and phosphorus absorption, triggers FGF23 synthesis and renal klotho expression and blocks PTH production.

CKD is strongly associated with mineral abnormalities leading to defective bone mineralization. Progressive decline in renal function reduces renal phosphorus clearance and calcitriol synthesis, which in turn, directly stimulate PTH secretion (40). Hypocalcemia, secondary to decreased calcitriol-induced calcium intestinal absorption, calcium-phosphate precipitation in extra-skeletal tissues and skeletal resistance to PTH action, further enhances and sustains secondary hyperparathyroidism (41). Current clinical data indicate that FGF23 rise precedes increments in PTH during the course of CKD (42). In fact, increased phosphate levels boost FGF23 production, which additionally aggravates calcitriol suppression, leading to further stimulation of PTH production (40). Furthermore, renal klotho expression is reduced in parallel with progression of CKD, which according to two hypothetical scenarios, may either precede serum FGF23 rise, leading possibly to target-organ resistance to FGF23 and maintenance of increased serum FGF23 levels, or may be secondary to negative feedback from primary FGF23 excess (42).

## Effects of Adipokine Profile on Mineral Homeostasis

### Results From Experimental Studies

**Figure 1:**
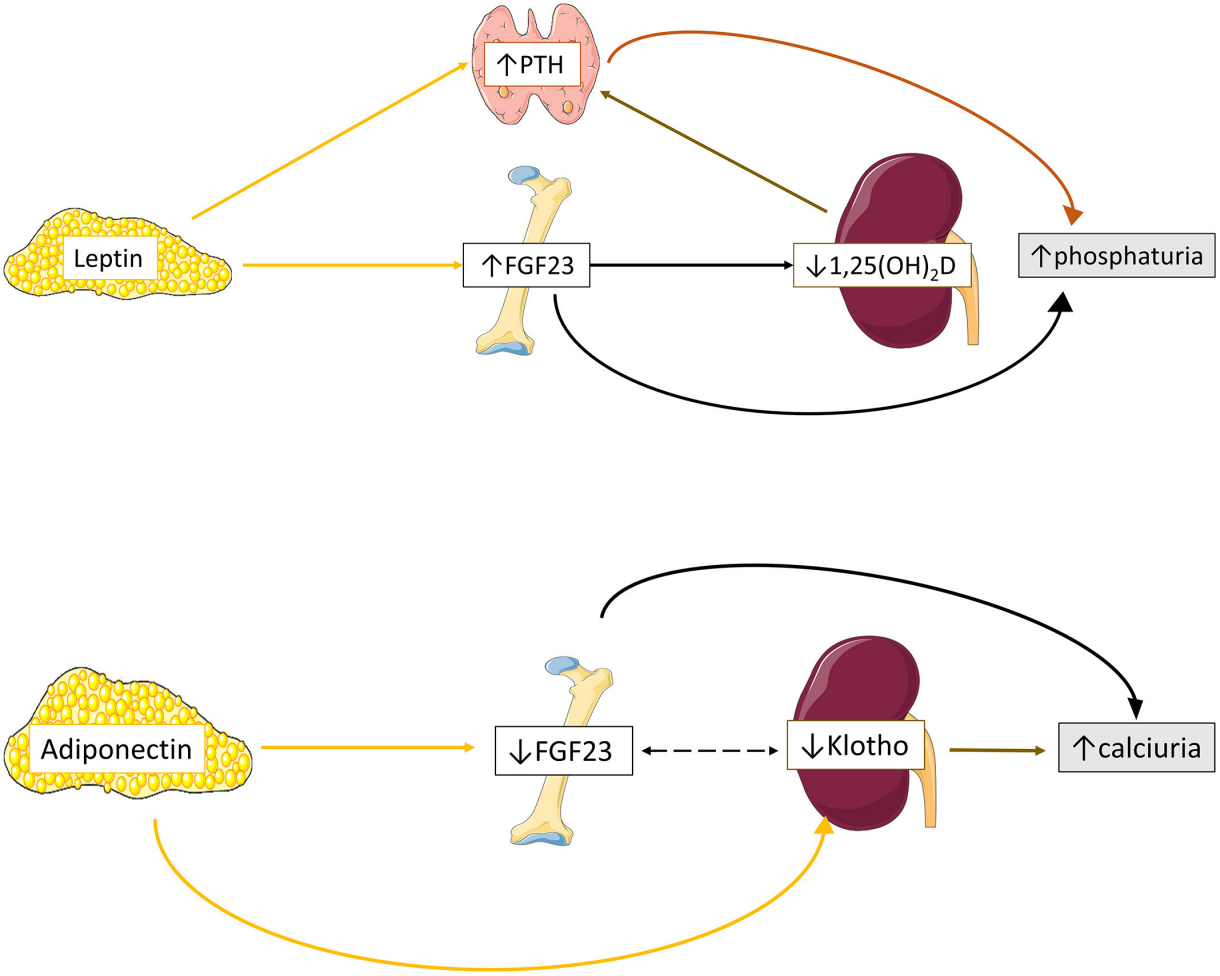
Proposed schematic overview of adipokines effects on mineral homeostasis. Leptin upregulates bone FGF23 expression leading to reduced 1,25(OH)_2_D production and subsequently to enhanced PTH excretion. In parallel, leptin directly stimulates PTH excretion. Both elevated FGF23 and PTH secretion ultimately results in increased phosphaturia. Adiponectin downregulates bone FGF23 and renal Klotho expression, leading to increased calciuria. Data from (11, 43–45).

Our current knowledge regarding the effects of adipokine profile on mineral homeostasis partially derives from experimental *in-vitro* and *in-vivo* studies (11) (Figure 1). We will focus on the reported impact of adipose tissue derived leptin and adiponectin on mineral metabolism.

#### Effects of Leptin on Mineral Metabolism

Tsuji et al. were the first to demonstrate that leptin administration directly stimulates bone FGF23 but not renal Klotho transcription in leptin-deficient mice (43), probably through activation of JAK-STAT pathway, leading to downregulation of type II sodium phosphate tubular cotransporter (NPT2a) and phosphaturia (43). Interestingly, in another study on osteocyte-like bone cells leptin administration lacked direct influence on FGF23 expression but positively modulated calcitriol induction of bone FGF23 production (46). The positive effect of leptin on FGF23 expression has shed light regarding its impact on vitamin D metabolism. *In-vivo* studies on leptin deficient mice have shown that upregulation of bone FGF23 secretion by leptin administration blocks proximal tubular calcitriol production, leading to reduction of serum calcitriol levels and vitamin D 1-a hydroxylase activity (43, 46–48).

The effects of leptin on PTH secretion has been examined in few experimental models (44). Leptin administration increased PTH expression in animal (49) and human (50) incubated parathyroid glands and in leptin-deficient and wild-type mice (43, 47, 49, 50). An indirect effect of leptin on parathyroid glands was suggested by Matsunuma et al. and Tsuji et al. studies, through upregulation of FGF23 expression (43) and downregulation of calcitriol production (43, 47). Nevertheless, Lopez et al. showed that leptin administration increased PTH levels, although no significant change in calcitriol and FGF23 expression was observed (49). Subsequently, leptin receptors were detected in the cytoplasm of both animal (49) and human parathyroid chief cells (50), suggesting a direct positive modulatory role of leptin on PTH secretion. Moreover, the detection of leptin expression on parathyroid gland tissue, and especially in case of hyperplasia, indicated that parathyroid gland is also a source of leptin production (50).

#### Effects of Adiponectin on Mineral Metabolism

Adiponectin seems to exert an opposite to leptin effect on FGF23 metabolism (45). According to Rutkowski, adiponectin inhibited osteocyte FGF23 and renal tubular epithelial Klotho expression *in vitro*, with no effect regarding to NPT2a transcriptional regulation (45), resulting in reduced serum FGF23 and Klotho levels and increased tubular fraction calcium excretion in adiponectin-overexpressing mice (45). Interestingly, although no change on PTH and calcitriol expression was observed *in-vivo* among adiponectin overexpressing, knockout and wildtype mice, adiponectin seems to modulate their expression after phosphorus and calcium loading (45). In detail, phosphorus loading resulted to relatively attenuated PTH in adiponectin knockout mice and amplified FGF23 release in adiponectin-overexpressing mice, while calcium loading boosted calcitriol expression in the latter type of mice (45).

### Impact of Obesity on Mineral Metabolism in General Population

The impact of obesity on mineral profile has been intensively studied during the last decades in various populations. Earlier studies were focused on patients with morbid obesity, in whom serum 25(OH)D levels were lower and serum PTH levels higher compared to healthy controls (51–54). Subsequent studies in healthy population confirmed that increased body mass index (BMI) is associated to hypovitaminosis D, while body fat mass is inversely correlated to serum 25(OH)D levels and positively correlated to serum PTH levels in both adult (55–61) and pediatric subjects (62–68). Furthermore, weight loss was accompanied by increase in serum 25(OH)D levels (69, 70) and decrease in serum PTH levels (70, 71) in both adult and pediatric cohort studies, suggesting that obesity is a modifiable risk factor of mineral disorder.

### Impact of Obesity on Mineral Metabolism in CKD

Along with clinical observations in general population, serum 25(OH)D deficiency was more prevalent in adult and pediatric overweight patients with CKD in various clinical studies (72–78) (Table 1). Moreover, obesity was positively associated with secondary hyperparathyroidism in adult patients with CKD (79–82) and in pediatric kidney transplant recipients (83), while low PTH level was considered as a marker of malnutrition-inflammation complex condition in CKD 5D patients (82) (Table 2). However, no association was observed between PTH and BMI status in other cross-sectional studies (75, 84).

**Table 1:** Results of clinical studies investigating the association between serum 25(OH)D levels, fat mass and adipokine profile in patients with chronic kidney disease (CKD).

**References**	**No patients**	**CKD stage**	**Results**
Figuiredo-Dias et al. (72)	120	35+/−15 ml/min	• Serum 25(OH)D was negatively correlated to serum leptin (*r* = −0.20, *p* = 0.027) and to subcutaneous abdominal fat (*r* = −0.23, *p* = 0.012). • BMI > 30 kg/m^2^ was a risk factor of hypovitaminosis D [25(OH)D <30 ng/ml] (OR 4.3, 95% CI 1.21–5.3, *p* = 0.018).
Barreto Silva et al. (73)	244	<60 ml/min	• Serum 25(OH)D was negatively correlated to serum leptin (*r* = −0.19, *p* = 0.03). • Total body adiposity >34.4% measured by DXA was independently associated with vitamin D deficiency [25(OH)D <20 ng/ml] (OR 2.3, 95% CI 1.1–5, *p* = 0.03). • BMI ≥ 25 kg/m^2^ and waist-to-height ratio>0.55 were not significantly associated with vitamin D deficiency (OR: 1.68, 95% CI 0.9–3.3, *p* = 0.13 and OR 1.41, 95% CI 0.7–2.9, *p* = 0.35, respectively).
Petchey et al. (74)	593	CKD stages 1-5	• A negative correlation was observed between serum 25(OH)D and BMI (*r* = −0.22, *p* < 0001). • On closer examination, the relationship between BMI and serum 25(OH)D was not linear but quadratic with both extremes of weight associated with lower serum 25(OH)D. • Normal BMI (18.5–24.9) was independently associated with vitamin D sufficiency [25(OH)D ≥ 30 ng/ml] (OR 1.94, 95% CI 1.22–3.07, *p* = 0.005).
Kitsos et al. (75)	159	>15 ml/min/1.73 m^2^	• Compared to normal-weighted patients, serum 25(OH)D was lower in overweight/obese patients in case of GFR ≥ 60 ml/min/1.73 m^2^ (*p* = 0.005), 30 ≤ GFR ≤ 59 ml/min/1.73 m^2^ (*p* = 0.001), and 15 ≤ GFR ≤ 29 ml/min/1.73 m^2^ (*p* = 0.030). • An independent negative correlation was observed between BMI > 25 kg/m^2^ and serum 25(OH)D (β −0.375, 95% CI −7.447–3.353, *p* < 0.001).
Baxmann et al. (76)	100	KTx	• Patients with either vitamin D deficiency [25(OH)D 15–30 ng/ml] or insufficiency [25(OH)D <15 ng/ml] presented higher body fat and weight gain since KTx (*p* < 0.001 and *p* < 0.001, respectively). • Waist circumference (*p* = 0.044), BMI (*p* = 0.001) and serum leptin (*p* = 0.001) were higher in patients with vitamin D deficiency. • Body fat (*r*^2^ = 0.366, *p* < 0.001) and serum leptin (*r*^2^ = 0.285, *p* < 0.001) were negatively correlated to serum 25(OH)D.
Seeherunvong et al. (77)	258	106 +/– 51 ml/min/1.73 m^2^	• BMI>85th perc was significantly more prevalent in patients with either vitamin D deficiency [15 ≤ 25(OH)D <30 ng/ml] or insufficiency [25(OH)D <15 ng/ml] (*p* = 0.02).
Kovesdy et al. (78)	978	KTx	• Serum leptin ≥ 15 μg/L was associated to higher 25(OH)D levels (*p* < 0.01).

*BMI, body mass index; GFR, glomerular filtration rate; KTx, kidney transplantation*.

**Table 2:** Results of clinical studies investigating the association between parathormone (PTH) and fat mass in patients with chronic kidney disease (CKD).

**References**	**No patients**	**CKD stage**	**Results**
Kitsos et al. (75)	159	GFR>15 ml/min/1.73 m^2^	• PTH levels were similar in obese/overweight vs. normal-weight individuals (*p* = 0.765). • No correlation was observed between BMI > 25 kg/m^2^ and PTH (β = 0.011, 95% CI −24.957–28.684, *p* = 0.891).
Ishimura et al. (79)	590	CKD 5D	• PTH was significantly correlated to fat mass (*r* = 0.171, *p* = 0.0014) in male but not in female patients (*r* = 0.109, *p* = 0.0885). • An independent correlation was observed between PTH and body weight (β = 0.190, *p* < 0.0001), BMI (β = 0.177, *p* < 0.0001) and fat mass (β = 0.142, *p* < 0.0005).
Kovesdy et al. (80)	496	GFR: 31.8 +/– 11.2 ml/min/1.73 m^2^	• PTH was independently associated with higher BMI (*p* = 0.008). This association was limited to patients with lower albumin (*p* = 0.005 for the interaction term) or higher white blood cell count (*p* = 0.026 for the interaction term).
Drechsler et al. (81)	1,628	CKD 5D	• PTH levels were lower in underweight, followed by normal weight, overweight and obese patients. • A ≥5% decrease in BMI over 3 months was associated with 26% decrease in PTH [PTH (ratio) 0.74, *p* = 0.039], whereas a ≥5% increase in BMI was associated with an 11% increase in PTH [PTH (ratio) 1.11, *p* = 0.026]. • Patients with PTH reduction and weight loss presented a 2-fold higher mortality (HR 2.02, 95% CI 1.45–2.83, *p* < 0.001), in contrast to those with decreasing PTH without weight loss.
Dukkipati et al. (82)	748	CKD 5D	• Lower ranges of PTH (<300 pg/mL) were correlated with the malnutrition-inflammation score (*r* = −0.17, *p* < 0.001), • A moderately low serum PTH in 100–150 pg/mL range was associated with the greatest survival compared to other serum PTH levels (HR 0.52, 95% CI 0.29–0.92, *p* < 0.001, compared to PTH of 300–600 pg/mL)
Vanderstraeten et al. (83)	149	KTx	• BMI SDS (β = 0.509, 95% CI 1.122–2.468, *p* = 0.011) was associated with hyperparathyroidism 12 months after KTx.
Marchelek-Mysliwiec et al. (84)	52	GFR: 15–60 ml/min/1.73 m^2^	• No significant correlation was observed between PTH and body fat mass (rs = 0.22, *p* = 0.11)
Peters et al. (85)	12	CKD 5D	• PTH was inversely correlated to total body fat (*r* = −0.69, *p* < 0.05) before parathyroidectomy (PTX). • Mean weight, BMI, conventional bioelectrical impedance measurements, total body fat, lean body mass and total body water were unaffected by surgery. • Phase angle and reactance significantly increased after PTX (*p* = 0.030 and *p* = 0.020, respectively).
Jiang et al. (86)	209	CKD 5	• BMI did not differ between patients with or without parathyroidectomy (PTX) (*p* = 0.128). • Successful PTX led to an increase on body weight (*p* = 0.025), BMI (*p* = 0.035), serum total cholesterol (*p* = 0.004) and triglycerides (*p* = 0.032) levels.

*BMI, body mass index; CKD 5D, chronic kidney disease stage 5 on chronic dialysis; GFR, glomerular filtration rate; KTx, kidney transplantation; SD, standard deviation*.

### Pathogenesis of Obesity Related Mineral Disorders

#### Obesity and Vitamin D Deficiency

**Figure 2:**
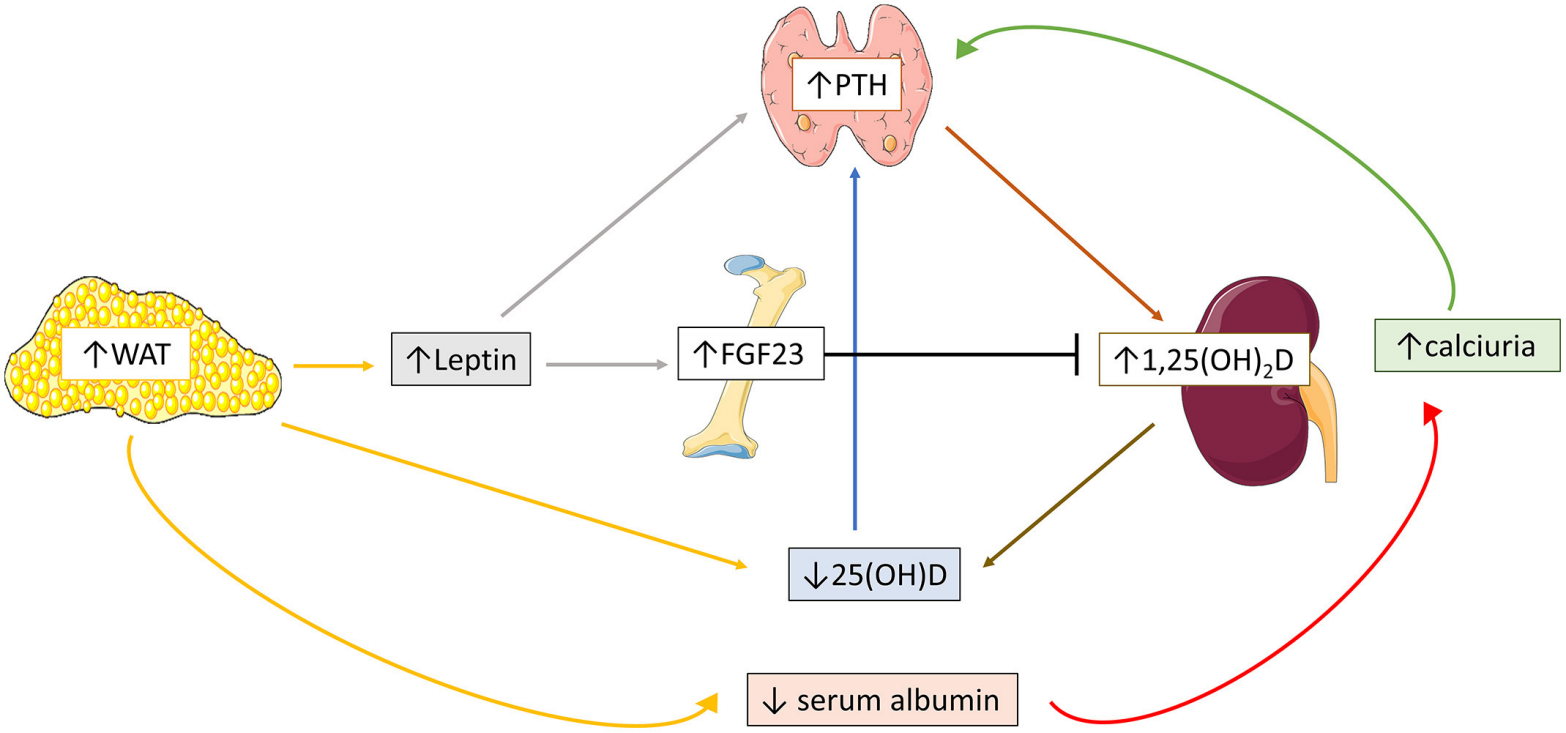
Schematic overview of the possible pathogenetic mechanisms implicated in obesity-related mineral disorders. Increased white adipose tissue (WAT) stimulates adipocyte leptin expression, which in turn triggers fibroblast growth-factor-23 (FGF23) and parathormone (PTH) secretion. High body fat mass is also associated with reduced serum 25(OH)D levels, which in turn accentuates PTH secretion. Increased 1,25(OH)_2_D expression secondary to high PTH levels further enhances 25(OH)D deficiency. Reduced renal tubular calcium reabsorption, due to decreased serum calcium-binding protein profile, additionally increases PTH expression.

Multiple pathogenetic mechanisms have been proposed for the comprised vitamin D status in obese population (Figure 2). Decreased bioavailability of vitamin D from cutaneous and dietary sources due to sequestration in body fat compartments (87–89) or volumetric dilution in the large fat mass (90) largely explains the lower serum 25(OH)D levels in obese population. Reduced sun ultraviolet B exposure, attributed to sedentary lifestyle, involving limited outdoor activities, and inadequate mineral intake from unhealthy high caloric food, may also play a role on the occurrence of hypovitaminosis D in obese individuals, who frequently require higher amount of cholecalciferol supplementation to normalize 25(OH)D levels compared to normal-weight controls (91–93). Nevertheless, in few adult and pediatric studies, the negative association between 25(OH)D and body fat mass remained significant after controlling for sunlight exposure and dietary intake of calcium and vitamin D3 (51, 94). Furthermore, according to some authors, lower serum 25(OH)D levels may be attributed to negative feedback from increased 1,25(OH)_2_D production, due to high serum PTH, on hepatic 25(OH)D synthesis (51). However, this assumption was not confirmed in all clinical studies. In specific, serum 1,25(OH)_2_D levels have been variously reported as higher (51, 54, 95, 96), similar (94), or lower (56, 97, 98) in obese population compared to normal-weight controls and the association between serum 25(OH)D and body adiposity status seems independent of serum PTH levels (58).

#### Obesity and Hyperparathyroidism

The pathogenesis of obesity-related hyperparathyroidism has been partly clarified (Figure 2). Hypovitaminosis D does not determinedly affect occurrence of obesity induced hyperparathyroidism, given that the association between serum PTH and body fat mass is independent of vitamin D status (54, 58). Some investigators suggest that the reduced calcium binding protein profile often encountered in obese subjects, may decrease renal tubular calcium reabsorption and serum ionized calcium, ultimately leading to hyperparathyroidism (71, 99). Nevertheless, the difference of serum ionized calcium between obese and normal-weight individuals was not always significant (51, 100). The absence of elevated serum PTH in non-obese vitamin D deficient subjects implies that adipose endocrine function modifies vitamin D—PTH axis (101). Recent clinical studies have shown that disrupted adipokine profile along with increased body fat mass is the main incriminating factor of disturbed mineral homeostasis. In specific, hyperleptinemia, commonly observed in obesity, was positively correlated to PTH in both adult and pediatric individuals (97, 102), indicating that stimulation of leptin—PTH axis is the initial source of hyperparathyroidism. Furthermore, increased serum leptin levels are probably implicated in the enhanced FGF23 expression in obesity. In detail, circulating FGF23 levels were often elevated in both adult and pediatric obese population, and positively correlated to fat mass accumulation (102–107). Of notice, non-remarkable changes in serum FGF23 levels were reported between hypertensive obese and non-obese children, while serum FGF23 levels were decreased in obese pediatric subjects in a cross-sectional study (108, 109). The conflicting impact of PTH and FGF23 on 1,25(OH)_2_D expression and *vice versa* may be the cause of the discrepancy of serum 1,25(OH)_2_D and FGF23 levels in obese population among different clinical studies (97).

## Effects of Mineral Homeostasis on Adipose Tissue and Adipokine Profile

### Results From Experimental Studies

#### Effects of Calcium on Adipose Tissue

During the last decades, there is emerging evidence favoring a key role of calcium balance on regulation of adiposity. Firstly, experimental studies demonstrated that agouti protein, an obesity gene product, acts on adipocytes *via* a calcium dependent mechanism, suggesting that adipocyte intracellular calcium regulates adipocyte lipid metabolism by stimulation of lipogenesis and inhibition of lipolysis (110). Calcium deficiency state promotes intracellular calcium overload, a state defined as calcium paradox (111). Toward this direction, enhancement of calcium intake in transgenic mice expressing the agouti gene reduced adipocyte calcium influx, and subsequently led to weight loss (110, 112). Further studies indicate that high-calcium intake disrupts gut fat absorption by promoting the formation of insoluble calcium-fatty acid soaps, which are ultimately excreted in the feces (113, 114), and may also negatively influence appetite (113, 115), or even activate calcium dependent apoptotic proteases in mature adipocytes (116), signifying a complex modulatory control of adiposity by calcium homeostasis (117).

#### Effects of PTH on Adipose Tissue and Adipokine Profile

The effect of PTH on adipose tissue remains controversial. It was generally hypothesized that PTH promotes fat storage, by enhancing calcium influx in adipocytes (118). Nonetheless, *in vitro* studies have shown that PTH induces lipolysis (119–121), probably *via* protein kinase A (PKA)-mediated phosphorylation of hormone-sensitive lipase (122). Further experiments in mice with primary hyperparathyroidism indicate that elevated PTH promotes browning of white adipose tissue, contributing to body weight loss (123). Toward the same direction, a recent study on CKD mice remarked that PTH contributes to white adipose tissue browning and wasting, by promotion of thermogenic genes expression, and more precisely uncoupling protein-1 (Ucp1), *via* PKA pathway activation, eventually leading to fat store depletion (124, 125).

The effect of PTH on leptin expression has not been clarified yet. In a study by Hoang et al., subcutaneous adipose tissue explants treatment with PTH resulted in increased leptin expression, indicating a positive modulatory role of PTH on leptin expression (50). Nevertheless, a negative feedback effect of PTH on leptin secretion was proposed in case of secondary hyperparathyroidism (44). Jiang et al. studied leptin and PTH interaction in case of secondary hyperparathyroidism by treating differentiated adipocytes *in vitro* with human serum belonging to CKD patients with severe secondary hyperparathyroidism before and after parathyroidectomy and to CKD patients with lower PTH levels (86). Adipocyte leptin expression and production was relatively reduced in case of severe secondary hyperparathyroidism and increased after parathyroidectomy (86). Furthermore, the authors found that high PTH levels suppressed adipocyte leptin production *in vitro via* inhibition of Akt signaling, indicating a negative effect of PTH on adipocyte leptin secretion (86). Conclusively, a positive loop between leptin and PTH is probably the case in primary hyperparathyroidism, whereas PTH seems to inhibit leptin expression in severe secondary hyperparathyroidism (44).

#### Effects of Calcitriol on Adipose Tissue and Adipokine Profile

The role of calcitriol on adipogenesis is probably equivocal, by either promoting (126) or impeding (127, 128) adipogenesis, depending of the type of adipocyte and stage of adipocyte differentiation (129). In specific, calcitriol treatment increases intracellular calcium and inhibits thermogenic gene uncoupling protein 2 (UCP2) expression in human adipocytes, leading to stimulation of lipogenesis and suppression of lipolysis (130, 131). On the contrary, calcitriol induces apoptosis of mature mouse 3T3-L1 adipocytes probably *via* activation of calcium-dependent calpain and caspase-12 (132). In CKD mice, intraperitoneal administration of calcitriol ameliorated cachexia, stimulated appetite, improved weight gain and fat mass content and attenuated the expression of thermogenic genes and other key molecules involved in adipose tissue browning (133).

The reciprocal effect of FGF23-calcitriol axis on adipokines secretion has not been elucidated yet. Long-term FGF23 deficiency does not affect fat metabolism in animal vitamin D receptor mutant mice (134) and FGF23 receptors are not present in adipose tissue (39), suggesting that FGF23 effect on adipose tissue is probably indirect, mediated by calcitriol induced activation of adipose tissue vitamin D receptor (135–137). The results of experimental models investigating the interplay between calcitriol and adipokine profile are contradictory and inconsistent. *In vivo* studies have shown that vitamin D receptor knockout mice develop a lean phenotype combined with lower serum leptin and higher serum adiponectin levels (138, 139), while mice with targeted overexpression of vitamin D receptor present obesity associated with higher serum leptin and lower serum adiponectin levels (140). Besides, direct stimulatory effect of calcitriol on leptin expression *via* a vitamin D receptor dependent manner was demonstrated in adipose tissue derived from mice epididymal fat pads (141). Accordingly, calcitriol attenuates adiponectin production in human pre-adipocytes (142). Nevertheless, *in vitro* studies have shown negative control of leptin secretion by calcitriol on human adipose tissue (143) and in mouse 3T3-L1 adipocytes (144). Furthermore, calcitriol treatment upregulated adiponectin in 3T3-L1 mature adipocytes (145) and in high glucose cultured 3T3-L1 adipocytes (146), while it had no effect on leptin expression in differentiated cultured human adipocytes in another *in-vitro* study (147). Taken together, there is a discrepancy regarding the role of calcitriol on leptin and adiponectin expression between *in-vivo* animal and *in-vitro* animal and human studies. Further studies are needed to enlighten the possible effect of calcitriol on the adipose tissue mass and adipokine profile depending on the type and maturation stage of target tissue.

#### Effects of Klotho on Adipose Tissue

Few experimental models have attempted to detect the role of Klotho on adipose tissue, through regulation of energy homeostasis. Administration of a-Klotho in obese high-fat feed mice resulted in reduced adiposity, increased lean mass, elevated energy expenditure and reduced lipid accumulation in liver and adipose tissue, probably by downregulating the expression of lipogenic genes (148). On the other hand, a-Klotho knock-out mice presented a barely detectable amount of white adipose tissue but preserved brown adipose tissue, reduced energy expenditure, mimicking a food-restricted condition (149). Moreover, a-Klotho suppression reduced while a-Klotho overexpression increased mRNA expression of adipocyte differentiation markers *in vitro*, suggesting that this hormone promotes adipocyte differentiation during the period of transient proliferation in the differentiation process (150). Further studies are needed to enlighten the potential role of a-klotho on fat metabolism.

### Impact of Mineral Homeostasis on Fat Mass and Adipokine Profile in General Population

The potential role of mineral homeostasis, including PTH, calcium and vitamin D status, on body fat mass in humans has been thoroughly investigated. It is current knowledge that patients with primary hyperparathyroidism usually exhibit higher BMI compared to healthy controls (151). Nevertheless, parathyroidectomy does not seem to determinately influence lipid profile and cardiovascular outcome of these patients (152, 153). On the other hand, according to a recent large-scale longitudinal case-control study, parathyroidectomy seems to finally lead to increased truck fat mass (152). Therefore, it is probable that obesity predisposes to the incidence of primary hyperparathyroidism, but it is unlikely that obesity is the result of the PTH anabolic effect on adipose tissue (151).

Although weight loss is associated with increased circulating 25(OH)D levels (69, 70), the reciprocal beneficial effect of vitamin D status on body fat mass remains controversial among clinical studies. Some clinical trials have reported that native vitamin D supplementation favorizes weight loss, body fat mass reduction and metabolic profile in obese adult (154) and pediatric (155, 156) population, while in other studies no related difference on weight loss was observed in obese subjects assigned to receive daily native vitamin D (157). Therefore, the effect of native vitamin D supplementation on successfully body fat mass reduction remains unclear, with a total insignificant effect reported by some (158, 159) but not all meta-analysis studies (160).

Emerging clinical data remarking a significant association between lower calcium intake and greater fat mass in both adult and pediatric population have raised the question whether calcium supplementation may facilitate weight loss in obese subjects (113). Despite the encouraging results of several trials indicating that higher calcium intake results in relatively lower fat mass gain (161), increased fecal fat excretion (162, 163), and ultimately fat loss (164), no significant effect was evoked in meta-analysis studies in both adult (165), and pediatric (166) subjects. Calcium combined to vitamin D supplementation seems a promising therapeutic option for facilitating weight loss, according to some trials, but data are still limited (167, 168). Conclusively, whether supplementation of both calcium and vitamin D may favorize weight loss and body fat mass reduction in subjects with initially low calcium diet and vitamin D deficiency as well as the supplementation dose required to achieve such outcome needs further investigation.

### Impact of Mineral Homeostasis on Fat Mass and Adipokine Profile in Patients With CKD

Clinical studies investigating the effects of mineral balance on fat mass and adipokine profile in patients with CKD are limited and principally concern adult population. We present the current relevant literature, and we suggest the possible mechanisms involved in the disturbed fat metabolism in the setting of CKD-related mineral disorders.

#### Effects of Secondary Hyperparathyroidism on Fat Mass and Adipokine Profile in Patients With CKD

According to Peters et al., severely increased serum PTH levels in CKD 5D patients with need for parathyroidectomy exerted a negative effect on total body fat mass (85). Moreover, successful parathyroidectomy led to improvement of malnutrition and increase in weight and BMI in adult patients with severe secondary hyperparathyroidism (86). Therefore, although obesity *per se* may be associated with higher serum PTH levels in CKD patients, severe secondary hyperparathyroidism seems to be inversely correlated to body adiposity status and implicated in the pathogenesis of advanced CKD-related cachexia (169). These results are in accordance with the white adipose tissue browning effect of secondary hyperparathyroidism observed in CKD mice (124, 125).

Taking into consideration that adipokine profile majorly reflects body fat mass levels even in patients with CKD, the discrepancy among the reports investigating the association between PTH and fat mass may also explain the variety of associations observed regarding the correlation between PTH and adipokine profile in this population (29, 44). In detail, serum leptin was negatively correlated to PTH in three clinical studies including 46, 73, and 161 hemodialysis patients, respectively (25, 170, 171), confirming the negative impact of secondary hyperparathyroidism on leptin secretion remarked in experimental studies (44, 86). However, leptin was positively correlated to circulating PTH levels in a cohort of 978 kidney transplant recipients (78) and 142 patients with CKD stage 2–5 (172), while no correlation was observed in three cross-sectional studies of 37, 72, and 107 hemodialysis patients (173–175). Interestingly, serum adiponectin levels were positively correlated to circulating PTH levels in a large scale cross-sectional study, including 716 patients with various CKD stages (176). This result accords with the findings of lessened PTH secretion in adiponectin knockout mice after phosphate loading (45). Considering that phosphate retention is increased in CKD, higher adiponectin levels are expected to be correlated to higher PTH levels.

**Figure 3:**
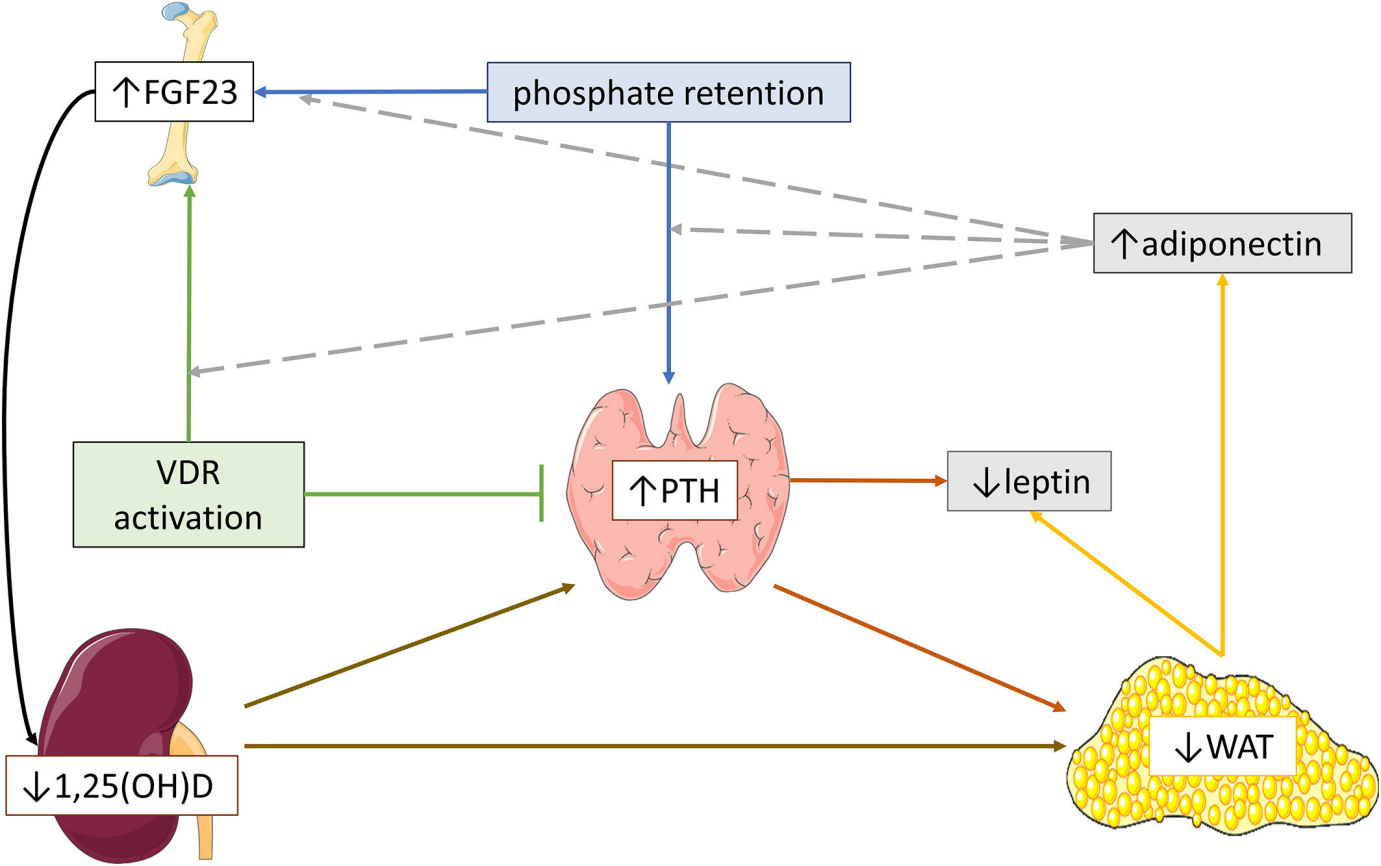
Schematic hypothetical overview of the possible pathogenetic mechanisms implicated in the effects of parathormone (PTH) and fibroblast growth factor-23 (FGF23)-calcitriol axis on adipose tissue and adipokine profile in severe CKD. In advanced chronic kidney disease, severe secondary hyperparathyroidism and reduced 1,25(OH)_2_D expression may induce white adipose tissue (WAT) reduction, leading to lower serum leptin and higher serum adiponectin. In parallel, high circulating FGF23 levels might aggravate WAT reduction *via* blockage of 1,25(OH)_2_D expression, which in turn, further stimulates PTH secretion. Moreover, severe secondary hyperparathyroidism possibly downregulates leptin expression. Higher serum adiponectin may favorize the upregulation of FGF23 and PTH secretion by phosphate retention and the upregulation of FGF23 secretion by vitamin D receptor (VDR) activation.

Conclusively, according to clinical and experimental studies, higher body fat mass may promote secondary hyperparathyroidism, but simultaneously, severe secondary hyperparathyroidism may contribute to white adipose tissue reduction. Additionally, PTH possibly blocks leptin secretion in case of severe secondary hyperparathyroidism. Moreover, higher serum adiponectin levels seem to favorize PTH secretion, especially in the context of increased phosphate retention (Figure 3). Further studies are needed to draw firm conclusions regarding the potential role of the severity and duration of secondary hyperparathyroidism on the disturbed fat metabolism in CKD.

#### Effects of FGF23/Calcitriol Axis on Fat Mass and Adipokine Profile in Patients With CKD

The association between FGF23 and body adiposity status has been rarely studied in CKD. Circulating FGF23 levels were negatively correlated to BMI (177) and fat mass (178) in adult CKD 5D patients in two recent studies. There are some hypotheses that may justify these findings. Although higher body fat mass may enhance FGF23 secretion, excessive FGF23 expression may reflect the decreased calcitriol expression or the severe secondary hyperparathyroidism, which in turn, aggravate adipose tissue metabolism leading to reduced fat mass (124, 125, 133) (Figure 3). Nevertheless, no correlations were observed between body weight, BMI or fat mass and serum FGF23 levels in early and moderate CKD in both adult (84) and pediatric (179, 180) patients (Table 3). The reasons behind the discrepancy among the results of different studies might be attributed to the variable FGF23 release, which rises along with the severity of CKD, but additional studies are required for further clarification.

**Table 3:** Results of studies investigating the association between fibroblast growth factor-23 (FGF23) levels, fat mass and/or adipokine profile in patients with chronic kidney disease.

**References**	**No. of patients**	**CKD stage**	**Results**
Montford et al. (177)	654	CKD 5D	• An increase per SD in log10 c-terminal FGF23 was independently associated with lower BMI (β = −1.11, *p* = 0.008), TC (β = −6.46, *p* = 0.02), LDL-C (β = −4.73, *p* = 0.04), and HDL-C (β = −2.14, *p* = 0.03).
Chiang et al. (178)	611	CKD 5D	• C-terminal FGF-23 was independently and inversely associated with BMI (−0.24 kg/m^2^ per 50% higher FGF-23, 95% CI −0.38–0.10), WC (−0.44 cm per 50% higher FGF-23, 95% CI −0.79–0.08), and % body fat (−0.58% per 50% higher FGF-23, 95% CI: −0.79–0.37). • C-terminal FGF-23 was inversely associated with serum leptin in univariate analysis (−0.21 pg/mL per 50% higher FGF-23, 95% CI−0.29 –0.14). • C-terminal FGF-23 remained significantly associated with %body fat after adjustment for serum leptin levels (−0.17% per 50% higher FGF-23, 95% CI −0.32 –0.02).
Sgambat et al. (179)	593	GFR: 30–90 ml/min/1.73 m^2^	• Participants classified as overweight had similar circulating c-terminal FGF23 levels to lean patients.
Bacchetta et al. (180)	227	GFR: 98 +/– 34 ml/min/ 1.73 m^2^	• No correlation was observed between FGF23 (intact and C-terminal) and relative height and body weight (expressed as SD score).
Spoto et al. (181)	88	CKD 3–4	• Intact FGF23 was independently and positively correlated to serum adiponectin (β = 0.22, *p* = 0.003). • Increase in FGF23 after paricalcitol treatment was substantially higher (*p* = 0.009) in the highest adiponectin quartile than in the other quartiles.
Marchelek-Mysliwiec et al. (84)	52	GFR: 15–60 ml/min/1.73 m^2^	• No correlation was observed between FGF23 and body fat mass (rs = 0.23, *p* = 0.15), serum adiponectin (rs = −0.08, *p* = 0.35), and serum leptin (rs = 0.2, *p* = 0.14).
Hyun et al. (182)	1,435	Predialysis CKD Stages 1–5	• C-terminal FGF23 was positively associated with higher serum adiponectin (*p* < 0.001). • High FGF23 patient group presented increased risk of CAC (OR 1.97, 95% CI 1.10–3.53). The association between FGF23 and CAC was modified significantly by adiponectin level (*p* for interaction = 0.023).

*BMI, body mass index; CAC, coronary artery calcification; CKD 5D, chronic kidney disease stage 5 on chronic dialysis; GFR, glomerular filtration rate; HDL-C, high-density lipoprotein cholesterol; LDL-C, low-density lipoprotein cholesterol; SD, standard deviation; TC, triglycerides; WC, waist circumference*.

Results of studies investigating the association between FGF23 and adipokine profile are controversial. Both serum adiponectin levels and changes were positively correlated to corresponding serum FGF23 levels and changes in adult CKD patients (181, 182). Interestingly, serum leptin was negatively correlated to circulating FGF23 in an adult CKD 5D study (178). Nevertheless, no significant correlations between FGF23 and either serum adiponectin or leptin were observed in early and moderate CKD adult patients (84). Moreover, paricalcitol-induced stimulation of FGF23 release was amplified in patients with higher serum adiponectin levels, suggesting that adiponectin is a strong modulator of FGF23 response to vitamin D receptor (VDR) activation (181). Therefore, higher serum adiponectin levels possibly enhance FGF23 secretion, in the context of increased phosphate retention, similarly to the findings observed in adiponectin-overexpressing transgenic mice (45), or possibly after VDR activation (181) (Figure 3).

## Conclusions

In conclusion, there is emerging compelling evidence that fat and mineral metabolism are linked in both general population and CKD patients. Therefore, aiming for balanced fat mass and mineral homeostasis is crucial for optimal health of both systems. In general population, randomized controlled trials are necessary to target the optimal mineral status in order to impede or even facilitate reduction of obesity. In CKD state, prospective studies are needed to explore the impact level of increased adiposity on mineral disorders, as well as the impact level of severe mineral disorders on CKD-related fat loss.

## Author Contributions

VK and NP contributed to initial conception. VK, AC, and NP contributed to design. VK, AK, and JD contributed to literature review. VK contributed to writing of the manuscript. AK and JD contributed to revision of the manuscript. AC and NP contributed to final revision of the manuscript. All authors contributed to the article and approved the submitted version.

## Conflict of Interest

The authors declare that the research was conducted in the absence of any commercial or financial relationships that could be construed as a potential conflict of interest.

## Publisher's Note

All claims expressed in this article are solely those of the authors and do not necessarily represent those of their affiliated organizations, or those of the publisher, the editors and the reviewers. Any product that may be evaluated in this article, or claim that may be made by its manufacturer, is not guaranteed or endorsed by the publisher.
